# Detecting and Understanding Social Influence During Drinking Situations: Protocol for a Bluetooth-Based Sensor Feasibility and Acceptability Study

**DOI:** 10.2196/50650

**Published:** 2024-06-06

**Authors:** Kristina Jackson, Matthew Meisel, Alexander Sokolovsky, Katie Chen, Nancy Barnett

**Affiliations:** 1 Center for Alcohol and Addiction Studies, Department of Behavioral and Social Sciences Brown University Providence, RI United States; 2 Brown University Providence, RI United States

**Keywords:** Bluetooth technology, passive sensing, social influence, alcohol use, ecological momentary assessment, social network, feasibility, acceptability, mobile phone

## Abstract

**Background:**

High-risk alcohol consumption among young adults frequently occurs in the presence of peers who are also drinking. A high-risk drinking situation may consist of particular social network members who have a primary association with drinking. Fine-grained approaches such as ecological momentary assessment (EMA) are growing in popularity for studying real-time social influence, but studies using these approaches exclusively rely on participant self-report. Passive indicators of peer presence using Bluetooth-based technology to detect real-time interactions have the potential to assist in the development of just-in-time interventions.

**Objective:**

This study seeks to examine the feasibility and acceptability of using a Bluetooth-based sensor and smartphone app to measure social contact in real-world drinking situations.

**Methods:**

Young adults (N=20) who drink heavily and report social drinking will be recruited from the community to participate in a 3-week EMA study. Using a social network interview, index participants will identify and recruit 3 of their friends to carry a Bluetooth beacon. Participants will complete a series of EMA reports on their own personal Android devices including random reports; morning reports; first-drink reports; and signal-contingent reports, which are triggered following the detection of a beacon carried by a peer participant. EMA will assess alcohol use and characteristics of the social environment, including who is nearby and who is drinking. For items about peer proximity and peer drinking, a customized peer list will be presented to participants. Feedback about the study protocol will be ascertained through weekly contact with both index and peer participants, followed by a qualitative interview at the end of the study. We will examine the feasibility and acceptability of recruitment, enrollment of participants and peers, and retention. Feasibility will be determined using indexes of eligibility, enrollment, and recruitment. Acceptability will be determined through participant enrollment and retention, protocol compliance, and participant-reported measures of acceptability. Feasibility and acceptability for peer participants will be informed by enrollment rates, latency to enrollment, compliance with carrying the beacon, and self-reported reasons for compliance or noncompliance with beacon procedures. Finally, EMA data about peer proximity and peer drinking will support the validity of the peer selection process.

**Results:**

Participant recruitment began in February 2023, and enrollment was completed in December 2023. Results will be reported in 2025.

**Conclusions:**

The protocol allows us to examine the feasibility and acceptability of a Bluetooth-based sensor for the detection of social contact between index participants and their friends, including social interactions during real-world drinking situations. Data from this study will inform just-in-time adaptive interventions seeking to address drinking in the natural environment by providing personalized feedback about a high-risk social context and alerting an individual that they are in a potentially unsafe situation.

**International Registered Report Identifier (IRRID):**

DERR1-10.2196/50650

## Introduction

### Background

Alcohol use typically begins and escalates between the ages of 16 and 20 years, and prevalence rates for heavy drinking and related consequences are the highest among those aged between 18 and 25 years compared with all other age groups [1]. During this time of emerging adulthood, individuals spend approximately 10 hours per week socializing with their friends [2], and many of these interactions occur in the context of alcohol use. Young adult drinking most frequently occurs in a social context, in groups, and with close friends [3-5]. Moreover, youth who drink in social settings consume more alcohol than they do when drinking alone [6,7] and report more alcohol-related consequences such as interpersonal violence, risky sexual behavior, and driving under the influence of alcohol [8,9]. Thus, understanding how peers influence and interact with each other during drinking situations is of utmost importance.

### Influence of Peers on Drinking

Survey research demonstrates that regular exposure to peers who value alcohol use and support heavy drinking is associated with higher adolescent alcohol use [10] and that in young adulthood, the proportion of alcohol-consuming friends is concurrently and prospectively associated with an individual’s drinking [11-13]. The expansion of social networks during young adulthood and the increase in proximity and salience of same-age peers [14-16] may exacerbate increases in alcohol use during this time [17].

A high-risk drinking situation may consist of particular social network members who have a primary association with drinking. Influential peers, colloquially known as “drinking buddies,” have many traits that are characteristic of close friends, including frequent contact, relationships of long duration, and high levels of emotional and concrete social support [18,19]. The presence of these individuals in one’s social network confers additional alcohol-related risk, even after controlling for the number of drinkers in the network [20-22]. Heavy drinking could potentially be triggered by the presence of these influential peers in close proximity. The presence of influential peers could be a single key indicator of heavy-drinking situations. In addition, peers are a frequently cited source of alcohol [23,24] often by directly offering alcohol [25] or by pressuring others to drink [26].

### Examination of Drinking Contexts Using Technology

Ecological momentary assessment (EMA) involves repeated sampling of individuals’ behaviors and experiences in real time and in naturalistic settings [27]. As data are collected in situ, findings are more generalizable to real-world, real-life experiences and processes [27]. Data also are less prone to retrospection bias [28]. Individuals are better at recalling their cognitions and behavior across a day compared with weekly or monthly retrospective reports [29]. EMA reports show poor concordance with timeline follow-back assessments, and EMA data have been demonstrated to both overestimate [30] and underestimate [31] the number of drinks relative to timeline follow-back assessments. EMA designs also allow for the examination of changes in a behavior over time and across contexts and permit within-person comparisons. For example, researchers can test whether there are unique differences in the social context on days that a person drinks alcohol versus on days they do not drink alcohol. EMA has been used to examine situational predictors of drinking behavior, including social context (with whom) and physical context (where) [32-34]. However, there are 2 primary limitations of EMA when applying it to study peer influence. First, individuals must be actively aware of and be able to report about peer influence. Second, there is a significant bias toward recent and salient peer proximity when self-reported [35]. Owing to these 2 significant limitations, there is value in research that relies less on self-report and more on passive assessment.

App-based ambulatory assessment of behavior is on the rise [36,37], with smartphones collecting multiple types of data from built-in sensors in real time [38]. Examples of passive sensing in alcohol research include transdermal alcohol biosensors to continuously measure alcohol consumption [39,40] and heart rate variability to study associations with stress and anxiety [41]. EMA studies in the tobacco and cannabis fields have used geographic EMA methods to passively capture contextual changes as individuals move through their days [42-44]. These methods combine EMA with real-time mobile geographic location tracking technology obtained through GPSs and geographic information systems. A drinking report can be triggered when an individual enters a specific zone (a geofence), reducing the burden on the individual to self-initiate reports in this location. Thus, there is support for work that identifies and targets high-risk situations based on a link between a given context and substance use.

However, despite the well-established importance of peer influence on alcohol use, no extant studies have used passive assessment to understand the real-world social contexts of drinking. As a ubiquitous low-power connectivity feature embedded in mobile phones and many other wearable sensors, Bluetooth provides a possible method to study peer contact in settings in which peers are influential. Specifically, Bluetooth-based transmitters or beacons (eg, Apple AirTags or other transmitters including smartphones) have unique addresses that can be identified by Bluetooth receivers (eg, smartphones), and thus, Bluetooth beacons carried or worn by individuals can be used with smartphone apps to assess the duration and frequency of interpersonal interactions [45]. Only few studies have used Bluetooth sensors to study health behaviors [46,47], including COVID-19 spread [48-50], but to the best of our knowledge, passive sensor technology has not been used to investigate the social context of alcohol use.

### Study Objective

This study aims to evaluate the feasibility and acceptability of (1) using a social network interview (SNI) to identify influential drinking peers and (2) using a Bluetooth-based sensor for the detection of contact between heavy drinkers and their peers. In a companion paper, we have described the technology that uses Bluetooth-based sensors to detect social contact [51]. Together, these papers summarize the procedures for a study that will develop and test the utility of a Bluetooth-based sensor to detect contact between individuals in drinking studies. The methods for evaluating feasibility and acceptability are described in Table 1.

**Table 1:** The study’s primary goals and methods for evaluating feasibility and acceptability.

Participants and goals			Definition		Method of evaluation		Data source
**Index participant**							
	**Feasibility**						
		Proportion of screened respondents who are eligible based on the inclusion criteria		Number of eligible respondents among all respondents who completed the screening survey		Screening survey Indexes of consent and enrollment	
		Proportion of screened respondents ineligible due to mobile phone operating system		Number of respondents eligible based on the screening survey, excluding the reason of not having an Android phone		Screening survey Indexes of consent and enrollment	
		Time required to enroll index participants		Number of months from start of recruitment to reaching the desired sample size		Indexes of consent and enrollment	
		Duration between notification and survey completion		Time elapsed between signal-contingent report notification and EMA^a^ survey completion and submission		EMA report notification and data time stamps	
	**Acceptability**						
		Proportion of eligible index participants who enroll in the study and who participate in the study		Number of respondents who enroll in the study among all the eligible respondents Number of respondents who participate in the study among those who enroll (defined as the proportion of enrolled participants for whom eligible peers are identified and enrolled) Postscreening self-reported reasons for nonparticipation		Screening survey Indexes of consent and enrollment	
		Retention of index participants through the study protocol—defined as high retention rate at 90%		Number of index participants completing the study protocol among those who enrolled		Project tracking database Postparticipation interview	
		Index participants’ compliance with project procedures—defined as typical completion rate of EMA reports for substance use research—75% for signal-contingent and random reports [52]		Separate indexes of number of signal-contingent and random reports completed among those expected Total number of triggered signal-contingent and random reports will be considered to evaluate the impact of participant burden on compliance		EMA report notification and data time stamps Postparticipation measure of reasons for noncompliance	
		Index participant ratings of the functionality and utility of the EMA app		High ratings on system utility measures and few reports of barriers to use		EMA morning report of previous-day app errors Postparticipation measures (adapted System Usability Scale and interview)	
**Peer participant**							
	**Feasibility and acceptability**						
		Peer consent and enrollment rate		Proportion of eligible peers who consent Proportion of peers who consent but do not enroll Peer self-reported reasons for nonparticipation after providing consent		SNI^b^ Indexes of consent and enrollment	
		Peer retention		Number of peers completing the study protocol (completing the weekly surveys and end-of-study survey) among the peers enrolled in the study		Peer participant weekly surveys Postparticipation peer interview	
	**Acceptability**						
		Peer evaluation of protocol		Initial reactions to participation request, privacy concerns, and perceived impact on behavior or relationship with peer		Postparticipation peer interview	
		Compliance with beacon procedures, defined as the peer carrying the beacon with them throughout the study		Self-reported noncompliance, defined as the number of days and times when peer participants report not carrying the beacon Self-reported reasons for not carrying the beacon		Peer weekly surveys Postparticipation peer interview	
	**Feasibility**						
		Time required to enroll peers following identification by participant		Average time required to enroll first, second, and third peer		Indexes of consent and enrollment	
		Enrollment of 3 peer participants per index participant		Total number of eligible peers who enroll		Indexes of consent and enrollment	
		Bluetooth beacon return rate		Proportion of peer participants who return their beacon		Project beacon log	
	**Validity**						
		Successful identification of influential drinking peers		Proportion of participant drinking episodes in which peers identified during the SNI were present		SNI Project beacon log EMA report data	
		Successful selection of influential drinking peers to carry beacons		Proportion of drinking episodes in which a beacon-carrying peer was noted as present Proportion of drinking episodes in which a beacon-carrying peer influenced the participant’s drinking (offering alcohol or refilling a drink)		SNI Project beacon log EMA report data	

^a^EMA: ecological momentary assessment.

^b^SNI: social network interview.

## Methods

### Design

Young adults from the community will be recruited to participate in a 3-week EMA study with 3 peers. During the baseline session, participants will complete a battery of self-report measures and a research assistant (RA)–led SNI (described in the following sections) and will download a smartphone app. During the SNI, participants will identify influential peers, 3 of whom will carry beacons. Each day during the study, index participants will report about their own drinking behavior, which of their peers are nearby, and whether their peers are drinking using the smartphone app. At the end of the study, index participants will participate in a semistructured interview during which we will collect qualitative information, including feedback about their experiences in the study. Peers who carry sensors will complete weekly surveys and an interview at the end of the study to collect additional information about feasibility and acceptability.

### Participants

We will recruit 20 participants, anticipating that 15 (75%) will complete the data collection process. Participant inclusionary and exclusionary criteria include the following: (1) aged between 18 and 29 years; (2) a minimum of 1 nonsolitary drinking day per week, including 1 heavy-drinking episode (≥4 drinks for women and ≥5 drinks for men) per week in the past month; (3) able to read English; (4) own an Android smartphone with an operating system (OS) of version 11 or newer and carry it throughout the day; (5) have a data plan (limited or unlimited); (6) are willing to approach peers to participate; (7) not in or seeking treatment for substance use; and (8) no plans to travel for an extended period or other significant deviations in routine.

### Procedures

#### Eligibility and Recruitment

We will recruit young adults from the community, including both college-attending and non–college-attending young adults. Recruitment methods will include social media advertisements, flyers posted in retail establishments and on public streets, and notifications and email listserves at local universities. Advertisements will describe the study, including that participants will be asked to identify peers to be included in the study and will refer respondents to a brief Qualtrics screening survey, which will establish initial eligibility. Those who are eligible will be redirected to a separate survey where they will provide their contact information. Eligible individuals who decline to participate will be directed to an anonymous survey with a checklist of reasons for nonparticipation (eg, do not understand the study; do not think they have friends who would be willing to participate; Multimedia Appendix 1).

#### Participant Orientation and Baseline Assessment

An RA will schedule the 90-minute in-person baseline session, during which the index participants will complete the informed consent process, a brief battery of self-report measures, and the RA-led SNI. The RA will assist the index participant in downloading and logging into the EMA app and in adjusting the mobile phone settings and permissions. The RA will also demonstrate the EMA reports.

Participants will complete 4 types of EMA reports on their own personal Android devices via the PiLR platform application developed by MEI Research. *Signal-contingent* reports are triggered by the app following its detection of a beacon carried by a peer participant when a peer comes within approximately 15 feet from the index participant for at least 15 minutes. *Random* reports are intended to sample experiences throughout the day; index participants will receive 3 random reports per day between noon and midnight (distributed across 3 blocks of time: noon to 6 PM, 6 PM to 9 PM, and 9 PM to noon). *Signal-contingent* and *random* reports will have identical notifications in the app and will assess identical information, so that participants cannot differentiate between the two; both reports will assess alcohol use and the presence of and drinking by peers identified in the SNI. Participants will be instructed to complete these reports in response to notifications as soon as possible. *Morning* reports will be completed by participants every day, with the app providing a reminder at 10 AM. These reports will ask the participant about the previous day with items similar to those in the random and signal-contingent reports (ie, measuring who of their nominated peers they have spent time with and whether they have consumed alcohol). *Drinking event–contingent* reports will be initiated by participants when they start their first drink. Over the course of the study, study personnel will contact participants several times a week via SMS text message and as needed to check the progress, encourage compliance, and address technical issues. In addition, RAs will schedule weekly phone calls with participants to ensure that all issues and questions are addressed. We will ask participants to keep their mobile phone *on* and charged and not to change the mobile phone during the study period and will encourage the use of mobile phone pins or passwords.

After the 3-week EMA assessment is completed, we will conduct a 60-minute end-of-study qualitative phone interview with index participants to determine their reactions to participating in the study; any hesitation about participating; and their opinions about the EMA assessment, the Bluetooth beacon technology, and the peer selection process. Participants will also complete several end-of-study survey measures about their experience (Multimedia Appendix 1). We will assess whether the index participants changed any of their behaviors or interactions with others due to their participation in the study. Research staff will review the information about peer presence reported during the 3-week period with the participant. Index participants will be instructed about how to delete the app from their mobile phone at the end of the study, and their log-in credentials to the app will expire on their last day of data collection.

### Measures and Peer Selection

#### Baseline Survey

A web-based survey will assess sociodemographics including sex, gender, age, racial and ethnic groups, living arrangement, and college or employment status. *Alcohol consumption* will be measured using a series of items adapted from National Institute on Alcohol Abuse and Alcoholism [53] including past-year and past-month frequency of alcohol use and heavy alcohol use, typical quantity of use, maximum number of drinks, frequency of intoxication, and age of first use. *Alcohol consequences* and alcohol problems will be assessed using the Brief Young Adult Alcohol Consequences Questionnaire [54,55] and Alcohol Use Disorders Identification Test [56], respectively. A measure of *physical context,* stratified based on time of day (day, evening, or late at night), will assess drinking across the various locations (all that apply) with options such as your home (house, apartment, dorm, or residence hall), friend’s place, restaurant, bar, nightclub, and pub. A measure of *social context* (who do you drink with) will assess frequency (0=*never/almost never* to 4=*almost always/always*) in which the participant spends time (1) together without drinking; (2) together drinking; (3) together getting drunk; and (4) as a “drinking buddy” across various people such as roommates and housemates, romantic partner, boyfriend, or girlfriend, and classmates or coworkers. The frequency of *obtaining alcohol from various sources* will be assessed (0=*never* to 2=*frequently*), with options such as buy it at a store, available at a party, and someone gives it to me. Social norms will include *descriptive norms* (number of close friends and same-age peers who drink alcohol and get drunk monthly) and *injunctive norms* (perceived approval of drinking by a close friend and same-age peer) [57]. A 20-item measure of *drinking motives* assesses reasons for drinking alcohol related to coping, conformity, enhancement, and social reasons (0=*never/almost never* to 4=*almost always/always*) [58]. *Other substance use* (tobacco, Juul or vaping, marijuana, and illicit drugs) will be assessed. We will collect measures of *impulsivity* (18-item measure of sensation seeking [59,60]) and *drinker identity* such as “drinking is part of my self-image” (Alcohol Self-Concept Scale [61]).

#### SNI With Index Participants

The SNI entails asking the index participant to name up to 6 people of their age who are important to them. These people may be friends, family members, or anyone who they regularly spend time with in person [62,63]. Age, gender, residence (eg, do you live together) of network members, relationship type (eg, family or friend), and frequency of meaningful social interaction (“How often in a typical month do you spend at least 15 consecutive minutes with this person?”) will be collected. In addition, we will assess the alcohol use of the peer, including the following: (1) frequency of drinking (“How many times in the past month do you think this person drank alcohol?”) and (2) frequency of drinking with the peer (“In the past month, how often did you drink with this person [while both of you were drinking]?”). Finally, using a yes or no format, we will assess whether participants think the network member would be interested in carrying the beacon for the research study.

#### Peer Selection

During the baseline session, following the completion of the SNI, the RA will work with the index participants to identify 3 peers to ask to carry the beacon. The three inclusionary criteria for peers to carry the beacons are as follows: (1) aged between 18 and 29 years, (2) frequency of meaningful in-person social interaction is at least once a week in a typical week, and (3) drinking with the peer at least twice a month in a typical month. During the baseline session, with the assistance of the RA, the index participant will try to contact each of their selected peers via SMS text message, phone call, or video chat. If during the session, the index participant is able to contact the peer and the peer is interested in learning more about the study, the index participant will provide the contact information of the peer to the RA, and then, the RA will send the peer an authenticated Qualtrics link. The Qualtrics link will contain an explanation of the study and informed consent. If the peer does not respond to the index participant during the baseline session, the participant will send the peer a brief description of the study with the Qualtrics link. Peers who do not provide consent will be asked to complete a checklist indicating the reasons for declining to participate, which immediately follows the consent form (eg, “not enough time”; “do not trust that the research won’t be tracking them”; Multimedia Appendix 1). Index participants will be given 5 days to recruit a maximum of 3 peers to carry the beacons; participants who are unable to *identify* influential peers or are unwilling to contact peers in the orientation session will be withdrawn from the study. Index participants who are unable to *recruit* at least 1 peer to participate will not complete the EMA portion but will complete the end-of-study interview, as it collects information about the recruitment and peer selection process. If any peer who has been contacted has not responded to the participant within 3 days, we will inform the participant to contact the next peer on their SNI-identified list. The end goal is to have 3 peers who can carry the beacon, but we expect that we will need to make contact with >3 peers for some participants. To maintain confidentiality, index participants will not be directly told by the project staff which of their friends participated in the study. Peers who have declined to carry the beacon or were not asked to participate will still be listed as a peer in the EMA reports. Although we will not explicitly tell the index participant that they may participate in the full study with <3 peers, if we are unable to identify 3 peers or the peers are nonresponsive to the research team, at that point, we will inform them that they only need 1 peer to consent to participate for them to participate in the full study.

Once the set of SNI-identified peer participants have provided consent, each peer will be contacted by the project staff to schedule a time to receive the beacon and ask any questions. Each beacon is assigned a unique ID, which associates the beacon with the index participant and the peer. Peer names and unique beacon IDs will be uploaded into the EMA software, so that a customized list (maximum of 6 names) is presented to each participant for all EMA reports (asking whether any of their friends are nearby) and signal-contingent reports triggered by beacon detection can be associated with the correct peer. Peers on the list presented to the index participant may be carrying the beacon, or they may have declined to carry the beacon or not have been asked. This list can be updated in real time without involving the participant to avoid protocol disruptions.

The EMA reports will begin for index participants once the beacons are distributed to peers or within 5 days, whichever comes first. As up to 3 peers can be given a beacon, 3 signal-contingent reports could be triggered at a time (eg, if all the peers who were given a beacon come into contact with the participant at the same time); however, we do not anticipate that this will occur regularly. At the end of each week of data collection, peer participants will complete a brief web survey, and at the end of the study, they will complete a phone interview and return the beacon.

#### EMA Report Measures

**Figure 1:**
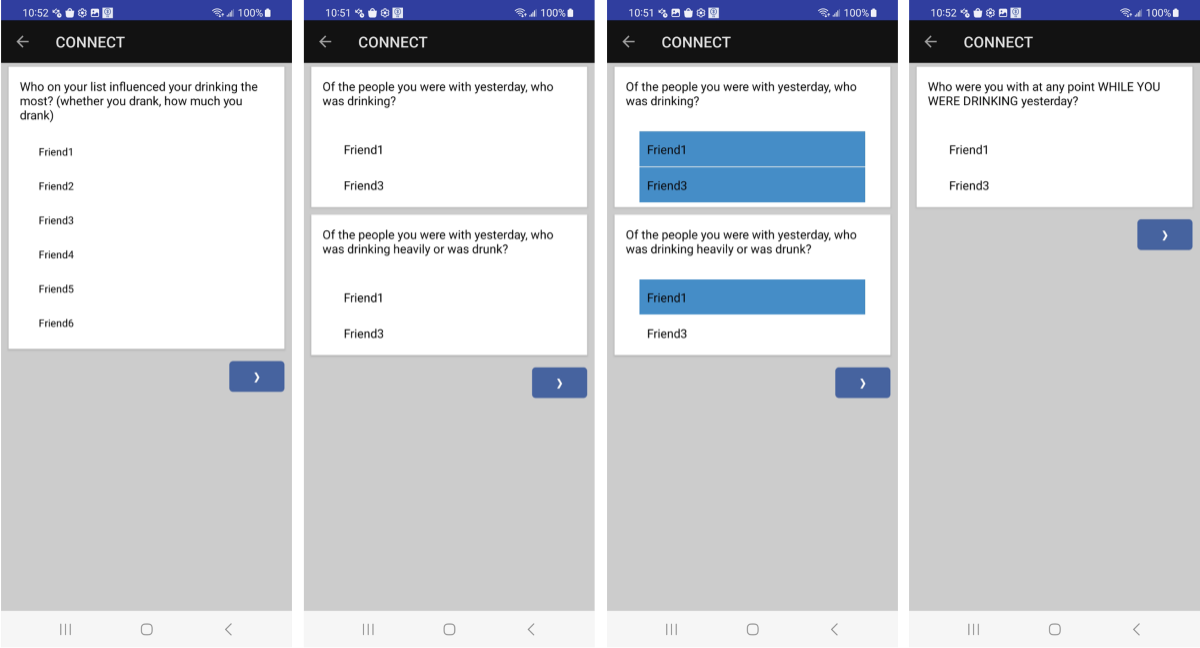
Survey flow.

The EMA measures are presented in Multimedia Appendix 1. The *random and signal-contingent reports* will appear identical on the EMA application to minimize unintentional awareness of peer presence. These reports will assess peer presence, participant drinking, and peer drinking and influence. *Index participant drinking* will be measured using questions about the number of drinks consumed, time at which drinking started and stopped, and level of perceived intoxication (“How intoxicated do you feel?”). Peer-related assessments will begin with a stem item measuring *peer proximity*: “In the past hour, who have you been around for any length of time?” The list of up to 6 most influential friends as determined in the SNI will be presented to the index participant, and they can select the names of their friends. Subsequent questions will present a revised list of friends according to which the friends were selected. Figure 1 presents screenshots of the sample survey flow. We will assess whether the index participant was within 15 feet from the peer for at least 15 minutes and whether they interacted with the peer in-person for at least 15 minutes. *Peer drinking and heavy drinking and drunkenness* will be measured using 2 items: “Of the people you were with in the past hour while you were drinking, who was also drinking?” and “Of the people you were with in the past hour, who was drinking heavily or was drunk?” *Direct and indirect peer influence* on participant drinking will be measured using questions about drink offers (“In the past hour, has anyone offered you a drink?”), suggestions to drink (“In the past hour, has anyone suggested that you should drink?”), and provision of alcohol to drinkers (“In the past hour, did anyone refill your drink or get you a new drink?”). Following affirmative answers, these 3 questions will be followed by determinations of whether the individuals selected were friends on the index participant’s peer list; if they are included in the peer list, the names of the peers indicated as present are shown.

The *morning report* will be identical to the signal-contingent and random reports but will refer to “yesterday,” whereas the signal-contingent and random reports will refer to “in the past hour.” Morning reports will also assess the total number of drinks taken on the previous day and the start time and end time of drinking. In addition, the morning report will include items measuring noncompliance and the mobile phone’s operational state as indicators of participant acceptability (Table 1) and possible reasons for missingness that could inform missing data analysis [64], including options such as silenced their mobile phone, turned off Bluetooth detection, mobile phone battery died, and mobile phone on battery or power saver mode (refer to Multimedia Appendix 1 for the full set of responses). We will also ask participants whether they did not complete a previous-day report because of their drinking, with response options such as no; yes, because I missed a notification; and yes, because I chose not to respond to a notification.

The *first-drink report* will focus specifically on alcohol involvement, including any drinking, heavy drinking, and drunkenness of peers. Again, we will query about people included and those not included in the peer list for specific actions, including offers of drinks. We will focus on the first drink because it is an index event that participants can identify and quickly answer questions about. We will not require additional momentary reports about that drinking event given that this information (including who was there at any time) is reported in the next-day morning report and we are cognizant of participant burden.

#### Peer Weekly Surveys

Peer participants will receive a link to a brief web-based survey at the end of each week. Peers will indicate days and time of day (day, evening, and late night) in the previous week they did not have their beacon with them, times in the previous week they spent time with their friend and drank with their friend, and whether they had difficulty in finding a place to keep the beacon or any problems in carrying the beacon with them.

#### Postparticipation Assessments

As part of our evaluation of acceptability, poststudy semistructured interviews will be conducted with index participants and peer participants. For index participants, we will solicit feedback about the peer recruitment process and the study protocol, including the frequency and timing of the EMA reports. Specifically, these interviews will ask participants about (1) identifying and inviting peers to participate, (2) using the app for answering EMA reports, and (3) appropriateness of contact from researchers and compensation for participating. Questions will also pertain to the usefulness of the technology from the participant’s perspective, including applications of the peer proximity technology to risky contexts (eg, heavy-drinking situations), and the usefulness of this technology for safety concerns in the context of heavy drinking. In addition, index participants will complete a modified 15-item System Usability Scale [65], with items adapted to our protocol that assess acceptability (eg, “I didn’t like that I had to change settings on my phone to use the app”). The System Usability Scale response options range on a 5-point Likert scale from 1=*strongly agree* to 5=*strongly disagree*.

For peer participants, we will collect the initial reactions to being asked to participate (reasons and concerns about participating), privacy concerns, adequacy of compensation, and perceived impact on the behavior of or relationship with the friend. We will also obtain feedback about the beacon, including ease of carrying the beacon with them every day and whether they would prefer to download an app in lieu of carrying the beacon. Multimedia Appendix 1 presents the full set of postparticipation assessments and peer measures.

### Ethical Considerations

Procedures were approved by the Brown University institutional review board (protocol 2022003448).

Index participants and peer participants will complete the informed consent process, which includes reviewing detailed consent forms, discussion with the researcher, and documentation of consent.

All information obtained during assessments will be confidential and used solely for research purposes. To protect data and prevent unauthorized access, all EMA data will be encrypted and will remain so until they are accessed by the project staff using a username and password specific to this project. All files with participant-identifying information will be password protected and stored separately from the data on a server accessible only to the project staff. To preserve confidentiality, we will deidentify data for both index and peer participants using a numeric code. Participants will have researcher-provided credentials for the smartphone app that will not include their names, and we will encourage participants to use mobile phone passwords.

As this protocol involves the passive assessment of social contexts and reporting about the behaviors of proximal others, considerable care was taken during the design phase to maximize confidentiality and increase participant comfort with responding. First, index participant reports inherently identify peers by presenting their names during daily reports. To preserve confidentiality, peer names are replaced by ID numbers in the analytic data sets and are thus unavailable for reidentification. Second, index participants’ discomfort associated with reporting about peer behavior will be minimized by ensuring that index participants understand that the smartphone app only records when it is within the range of the beacon and that no other information about their friend is recorded. We will protect peers against coercion by identifying eligible peers during the SNI but recruiting these peers without the presence of the index participant. To further reduce coercion, whether peers agreed to participate or were enrolled into the study will not be disclosed to the index participants unless the peer participant discloses this information themselves. It is possible that participants will identify which of their friends are participating in the study or will even encourage their friends to participate. We will also inform the peer participants that we will never share information about them or their weekly survey and interview responses with the index participant. To protect against peers’ discomfort about their friend’s smartphone app detecting their presence, we will clarify that no passively assessed information (eg, geolocation) other than peer presence is recorded.

Index participants will be paid US $50 for attending the baseline session, US $5 per day for answering the EMA reports, a weekly bonus of US $20 if they complete 80% of the random and signal-contingent reports, and US $40 for attending the exit interview. The highest compensation they can receive for participation is US $255 (compensation for peer participants is described below, following the section on peer selection). Peer participants will be compensated US $30 for each week they complete the weekly survey and interview and US $10 for returning the beacon to the research team. The highest compensation the peer participants can receive is US $100.

### Expected Results

Table 1 shows the list of project goals, how these goals are defined, the metric by which those definitions are evaluated, and the data source for each goal.

### Analytic Plan: Feasibility and Acceptability of Recruitment; Enrollment of Participants and Peers; Compliance; and Retention

The *feasibility* of recruiting index participants will be determined based on three indexes: (1) proportion of people who meet the inclusionary criteria among those who complete the screening survey, (2) proportion of screened respondents who are ineligible due to mobile phone OS (eg, iOS instead of Android), and (3) time required to reach the desired sample size. The *feasibility* of complying with the protocol is determined based on the time elapsed between receiving a signal-contingent report notification and EMA survey completion and submission. The *acceptability* of the procedures for index participants will be determined based on four indexes: (1) proportion of eligible individuals who enroll and participate in the study after reading a written explanation of the study—we will also calculate postscreening, self-reported mean ratings of reasons for nonparticipation and inspect each item as a measure of poor acceptability; (2) number of index participants completing the full study protocol compared to the number of index participants enrolled in the study; (3) proportion of completed reports among the total number of possible reports (morning and random reports only) and number of expected reports based on the project beacon log (signal-contingent reports only) and latency (in minutes) between triggering and completing a given report, calculated separately for random and signal-contingent reports (we will also examine whether compliance systematically varies as a function of timing and number of reports within a day); and (4) mean ratings on system utility measures and barriers to use, including ratings about the functionality and utility of the EMA app.

We will explore the *feasibility* and *acceptability* of recruitment and retention of peers. Feasibility will be determined based on three indexes: (1) total number of peers who are asked to carry the beacon, number of peers who provide consent, and number of peers who enroll, calculated across the full sample; (2) time required to recruit the first, second, and third peer by calculating the duration of time between baseline assessment and peer consent; and (3) whether 3 peer participants are successfully enrolled for each index participant. We will also informally explore best practices for enrolling peers with respect to timing (time required to make contact) and mode of contact (eg, text, call, or face to face). The feasibility of retaining peers will be determined based on the number of enrolled peers completing the study protocol (weekly surveys and end-of-study survey) among the number of peers enrolled in the study. Mean ratings of peer reasons for nonparticipation will be calculated similarly, and each item will be inspected.

The *feasibility* and *acceptability* of beacon procedures for peers will be determined based on two indexes: (1) number of days and times the peer carried the beacon with them and (2) proportion of distributed beacons that are returned to the study staff. Mean ratings of reasons for noncompliance with beacon procedures will also be calculated, and each item will be inspected.

### Analytic Plan: Validity of the Peer Selection Process

We will use 3 indications of whether (up to 6) influential peers were selected during the SNI and whether (up to 3) influential peers were selected to carry Bluetooth beacons, supporting the *validity* of our protocol. First, data from all EMA reports will be used to calculate the proportion of drinking episodes reported by the participant in which they reported that ≥1 of their listed peers were present. Second, we will calculate the proportion of drinking episodes in which the participant indicated that the present peers were also drinking. Third, when a participant indicates that someone (1) offered them a drink, (2) suggested they drink, (3) refilled their drink, or (4) influenced their drinking, the proportion of these peers who were included in the participants’ peer list (vs not included) will be computed. As these latter experiences could show low rates in some participants, we will combine them.

### Attrition, Nonresponse, and Missing Data

We will investigate person-level correlates of attrition for both index and peer participants by comparing the proportions of participants completing each week of the study (ie, days 7, 14, and 21) across baseline demographic and behavioral factors. We expect compliance with the study protocol to be consistent with current estimates (ie, approximately 75%; refer to the meta-analytic review across substance use studies using EMA designs by Jones et al [52]). Time-varying predictors of nonresponse will be examined within a conceptual framework that considers the impact of participant characteristics, protocol burden (eg, number of notifications received), and situational factors (eg, context and behavior) [66]. We will regress attrition or missingness onto these variables at their appropriate level of analysis and use the identified predictors of systematic missingness to inform future analyses to help maintain the missing-at-random assumption.

### Power

Although our sample size of 20 is similar to that of other pilot studies using EMA [67,68] and power to detect moderate-size, level-1 effects exceeds 0.80 (conservatively assuming 70% missingness and a binary grouping variable with an even split), we are likely underpowered for formal tests of moderation [69]. Instead, we will descriptively explore differences in SNI members’ presence in drinking events across sex (across all SNI members) and peer consent (across SNI members selected to carry beacons) by performing stratified analyses.

### Data Analysis and Dissemination

Data management and analysis will be conducted after all data have been collected, and findings will be reported in manuscripts submitted thereafter.

## Results

Participant enrollment began in February 2023. As of submission of the manuscript (June 2023), a total of 9 participants and 21 peers were enrolled. Data collection was completed in December 2023. Data analysis began immediately upon the completion of data collection. Results will be reported in 2025.

## Discussion

### Summary

In this paper, we describe a protocol that advances the literature by leveraging a newly developed Bluetooth-based detection system to understand influential social contact during real-time drinking situations. Understanding how young adults engage with and feel about this technology is important for determining whether just-in-time interventions using Bluetooth-based sensing can be implemented. Furthermore, evaluating whether the peers who are most influential on an individual’s drinking behavior can be identified and are willing and compliant in carrying a Bluetooth beacon is necessary in determining the feasibility of this protocol to identify situations where the index individual is most at risk.

### Study Implications

Most interventions aimed at reducing youth drinking are delivered distal in time to actual drinking events. Unfortunately, programs that neglect the socioecological context in which risky behaviors occur may have limited effectiveness, in part due to constraints on an individual’s ability to make sound decisions in a highly charged typically social context [70,71]. However, in recent years, there have been targeted efforts to reduce drinking in the natural environment, at a time when behavior is opportune for modification [72-76]. In just-in-time interventions, also referred to as ecological momentary interventions, individuals receive intervention components at specific moments throughout the day to increase health-promoting behaviors and reduce risky behavior [77-79]. However, although studies have identified effective just-in-time content across domains of health behavior [77], there is limited information about the appropriate timing or context in which to intervene.

Beckjord and Shiffman [80] have recommended using a combination of EMAs and ecologic momentary interventions where the delivery of real-time intervention is predicated on user-reported contextual data as well as passively recorded data to coincide with high-risk events. Some of the challenges with using self-reported EMA data as the antecedent for just-in-time intervention include the risk for systematic user noncompliance with EMA during high-risk events (eg, not reporting drinking events) and the need for burdensome EMA algorithm development, high use of devices (eg, geolocation leading to battery drain), and considerable monitoring by researchers, which may limit dissemination and impact [80]. Making just-in-time intervention content contingent on passive sensing overcomes many of these barriers and could facilitate timely delivery of intervention content that is less reliant on user interaction. Future interventions could incorporate Bluetooth-based sensor technology to help individuals who are trying to reduce their alcohol use. This technology could notify an individual through a smartphone or smartwatch that they need to be mindful of their alcohol intake. For example, personalized feedback could be provided when a “risky” peer is nearby and could alert the individual that they are in an unsafe situation, similar to smartwatch vibrations after a period of inactivity [81] or tailored messaging that are provided based on geolocation [82]. Our passive sensing approach could effectively complement geographic EMA by identifying both influential high-risk social contexts (peers) and high-risk physical contexts (locations). A friend or accountable individual could also be alerted that the participant is in a risky situation.

### Challenges and Limitations

There are several limitations in our protocol related to index participant behavior. It is possible that our use of a 15-minute criterion for a meaningful interaction will be inaccurate, resulting in, for example, us not detecting an influential encounter between a participant and a peer that is too brief to trigger a report. The decision to trigger signal-contingent reports after 15-minute interactions was based on a trade-off between sensitivity and burden. While meaningful social interactions <15 minutes that influence drinking may occur, triggering signal-contingent reports after shorter durations would yield more triggered reports, leading to potential disengagement (longer latency between trigger and completion of report) or noncompliance with the study protocol [52]. Although we plan to examine the relationship between burden and compliance in this protocol, future studies should systematically examine these trade-offs. We will query both behavior in the past 15 minutes and in the past hour in an attempt to accommodate delayed compliance.

Another potential limitation related to index participant behavior that is inherent to all substance use studies using ambulatory methods is the possibility that noncompliance is associated with the use of the substance itself. Participants may miss notifications when they are highly intoxicated [83]. Thus, we might expect alcohol use as reported at the survey level or day level to be associated with nonresponse. We will assess this potential reason for missing self-report data by examining the relationship between compliance with triggered or random reports and past-day drinking as reported in the morning report and self-reported reasons for missing a previous-day report as reported in the morning report. Intoxicated participants also may not recognize or record that a friend was present or influenced their drinking (eg, by offering alcohol or refilling a drink), resulting in underreporting of important peer interactions. Furthermore, there may be systematic noncompliance that is related to drinking context (eg, lower response rates when drinking in social situations such as loud parties or bars); this also can be explored using self-reported EMA data. However, one strength of this protocol is the capability to *passively* detect interactions with proximal peers without the need for self-report.

Technological limitations precluded the suspension of random reports after a signal-contingent report is triggered or vice versa. Thus, it is possible for both random and signal-contingent reports to be triggered around the same time, resulting in the index participant being notified to complete multiple identical reports. We expect this will happen infrequently. Although triggering of multiple signal-contingent reports may increase participant burden and increase the likelihood of nonresponse to a survey, detection of these unique social interactions by the smartphone app and completion of these reports will yield greater resolution of index participants’ social contexts and is critical to validate the overall protocol.

### Next Steps

This proof-of-concept study supports a long-term line of research characterizing the real-time social context of hazardous drinking, in turn informing the timing and context of mobile phone–delivered interventions to reduce hazardous alcohol use among young adults. Feasibility, acceptability, and initial substantive data will inform a large-scale study including participants with a wider age span and broader representation of social context, permitting the study of multiple dynamic determinants of drinking behavior including social and physical context, craving, affect, and motivation [84]. Passive peer detection can be used with other passive technology such as GPS (geolocation) for identification of localized risk, accelerometers for gait sensing, and ambient noise recording to evaluate the situational risk associated with social interactions. It can also be integrated with alcohol biosensors or other more objective measures of intoxication (eg, balance and gait assessed passively by the smartphone) to fill the gaps in reporting related to noncompliance or underreporting of alcohol use.

This protocol involves carrying Bluetooth beacons and requires Android OS smartphones, but future studies should explore the possibility for peers to download an app instead of carrying a beacon (which use the mobile phone’s Bluetooth feature to transmit a unique signal similar to the beacon) and to use Apple phones; however, this approach may be technologically limited by host OSs that restrict the development of features that duplicate extant commercially available products (eg, iOS and AirTags). Finally, although the proposed study targets peers associated with increased risk for alcohol use, future studies could also consider peers who are protective against heavy drinking. We hope that this proof-of-concept study stimulates future studies that leverage passive technology to understand the social nature of young adult drinking and ultimately reduce alcohol-related harms.

## Appendix

Multimedia Appendix 1 Assessment battery.

## Abbreviations

EMA: ecological momentary assessment
OS: operating system
RA: research assistant
SNI: social network interview

## Data Availability

Data sharing is not applicable to this paper as no data sets were generated or analyzed during this study. Once data are generated, data sets will be available from the corresponding author on reasonable request.
