# Supplementary material for: Detecting and Understanding Social Influence During Drinking Situations: Protocol for a Bluetooth-Based Sensor Feasibility and Acceptability Study
Source: JMIR Res Protoc. 2024 Jun 6;13:e50650. doi: 10.2196/50650 (PMC11190624; doi:10.2196/50650)
Supplement: Multimedia Appendix 1 [file resprot_v13i1e50650_app1.docx]

**Project Bluetooth Connect**

**EMA Assessment Battery**

**Morning Report**

INSTRUCTION: Please answer the following questions to the best of your ability.

1. Yesterday, who were you around for any length of time?
   - - [Person 1]
     - [Person 2]
     - [Person 3]

[Display IF #1 has Persons endorsed. Present only persons endorsed in #1]

1. Yesterday, who were you within 15 feet of for at least 15 minutes?
   - - [Person 1]
     - [Person 2]
     - [Person 3]

[Display IF #1 has Persons endorsed. Present only persons endorsed in #1]

1. Yesterday, who did you interact with in person for at least 15 minutes?
   - - [Person 1]
     - [Person 2]
     - [Person 3]

[Display IF #1 has Persons endorsed. Present only persons endorsed in #1]

1. Of the people you were with yesterday, who was drinking?
   - - [Person 1]
     - [Person 2]
     - [Person 3]

[Display IF #1 has Persons endorsed. Present only persons endorsed in #1]

1. Of the people you were with yesterday, who was drinking heavily or was drunk?
   - - [Person 1]
     - [Person 2]
     - [Person 3]


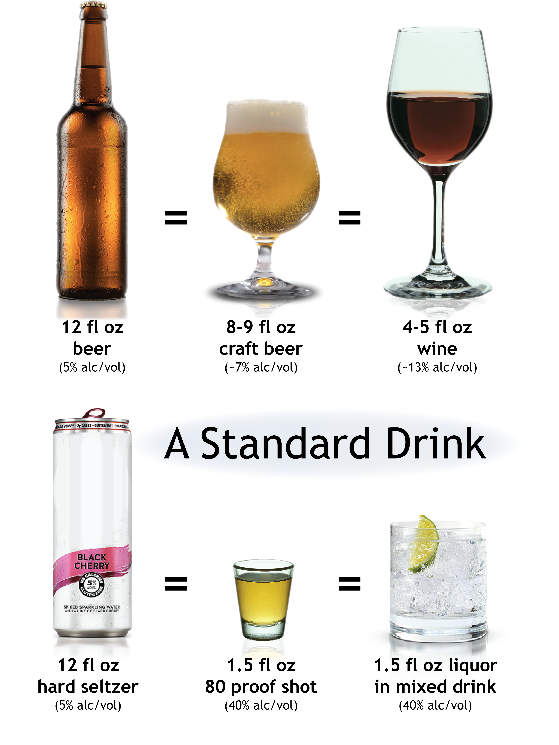


1. How many standard drinks did you have yesterday?

(forced choice 0-30)

[Display IF #6 >0]

1. What time did you START your FIRST drink yesterday? (time picker)

[Display IF #6 >0]

1. What time did you FINISH your LAST drink yesterday? (time picker)

[Display IF #6>0 AND IF #1 has Persons endorsed]

1. Who were you with at any point WHILE YOU WERE DRINKING yesterday?
   - - [Person 1]
     - [Person 2]
     - [Person 3]

[Display IF **#6**>0 AND IF #9 has Persons endorsed**]**

1. Who were you with when you started your FIRST drink yesterday?
   - - [Person 1]
     - [Person 2]
     - [Person 3]

[Display IF #6 >0 AND IF #9 has Persons endorsed]

1. Who were you with for at least 15 minutes WHILE YOU WERE DRINKING yesterday?
   - - [Person 1]
     - [Person 2]
     - [Person 3]

[Display IF #6 >0 AND IF #9 has Persons endorsed]

1. Of the people you were with yesterday WHILE YOU WERE DRINKING, who was also drinking?
   - - [Person 1]
     - [Person 2]
     - [Person 3]

[Display IF #6 >0 AND IF #12 has Persons endorsed]

1. Of the people you were with yesterday WHILE YOU WERE DRINKING, who would you say was drinking heavily?
   - - [Person 1]
     - [Person 2]
     - [Person 3]

[Display IF #6 >0 AND IF #9 has Persons endorsed]

1. Of the people you were with yesterday WHILE YOU WERE DRINKING, who would you say was drunk? (You might not have drank with this person)
   - - [Person 1]
     - [Person 2]
     - [Person 3]

[Display IF #6 >0 AND IF #9 has Persons endorsed]

1. Who were you with when you finished your last drink yesterday?
   - - [Person 1]

- [Person 2]
- [Person 3]

[Display IF #6 >0]

1. Yesterday, where were you when you consumed alcohol? (check all that apply)
2. Your home
3. Friend's place
4. Party
5. Bar/Restaurant
6. Outside
7. Sporting/entertainment event
8. Elsewhere

[Display IF #6 >0]

1. Did any of these happen due to your alcohol use yesterday? (check all that apply)
2. Felt nauseated/vomited
3. Was rude/obnoxious
4. Hurt/injured self by accident
5. Behaved aggressively
6. Said/did embarrassing things
7. Had a hangover
8. Forgot what you did (blackout)
9. Neglected school or work-related obligations
10. Drove a car after drinking
11. None of these happened

[Display to everyone]

1. Did anyone influence your decision to drink or not to drink yesterday? This could include influencing whether you drank, how much you drank, etc.

0) No

1) Yes

[Display IF #18 =1]

1. Who influenced your decisions about drinking yesterday? (you can check both answers)

1) Someone on my list

2) Someone NOT on my list

[Display IF #19 =1]

1. Who on your list influenced your drinking the most? (whether you drank, how much you drank)

[check one]

- - - [Person 1]
    - [Person 2]
    - [Person 3]

[Display IF #18 =1]

1. Please explain ways that anyone influenced your decision to drink or the way you drank yesterday.

[Open response]

[Display IF #6 >0]

1. Yesterday, did anyone do something to help you drink more safely (e.g., slow or stop drinking, avoid negative consequences, suggest that you get food)?

0) No

1) Yes

[Display IF #22 =1]

1. Who helped you drink more safely yesterday? (you can check both answers)

1) Someone on my list

2) Someone NOT on my list

[Display IF #23=1]

1. Who on your list did something to help you drink more safely?
   - - [Person 1]
     - [Person 2]
     - [Person 3]

[Display IF #22 = 1]

1. Please explain ways that someone did something to help you drink more safely.

[Open response]

[Display to everyone]

1. Was there a time yesterday that alcohol was available but you chose not to drink it?
2. No
3. Maybe
4. Yes

[Display IF #6 =0]

1. Do you think you will drink today?
2. No
3. Maybe
4. Yes

[Display to everyone]

1. If somebody offered you an alcoholic beverage later today, would you drink it?
2. Definitely not
3. Probably not
4. Probably yes
5. Definitely yes

[Display to everyone]

1. Did you use marijuana yesterday?

0) No

1) Yes

[Display IF #6 >0 OR #29 =1]

1. How intoxicated did you feel yesterday?

0) Not at all intoxicated

1) A little bit intoxicated

2) Moderately intoxicated

3) Very intoxicated

[Display IF #6>0 AND #29 =1 AND #30 = 1, 2, or 3]

1. How much of your intoxication yesterday was due to...

1) Only alcohol

2) Mostly alcohol, some marijuana

3) Equally alcohol and marijuana

4) Mostly marijuana, some alcohol

5) Only marijuana

[Display IF #29=1 AND #1 has Persons endorsed]

1. Who were you with while you were using marijuana? Include only people you were with in person.

- [Person 1]
- [Person 2]
- [Person 3]

1. Did you use any of these yesterday? (check all that apply)
   - - 1. E-cigarettes/vaping
       2. Cigarettes/cigars/cigarillo
       3. Nicotine pouch/snus/chew
       4. Other drugs
       5. None of the above
2. Did any of the following happen to you yesterday? (check all that apply)
3. Your phone battery died
4. You were away from your phone for more than an hour.
5. You couldn’t look at your phone (e.g., driving) so couldn’t see notifications.
6. You put your phone in “do not disturb” mode
7. You put your phone in Airplane mode
8. You turned on battery/power saver mode
9. None of the above
10. Did you do any of the following yesterday? (check all that apply)
11. You silenced your phone
12. You turned your phone off
13. You turned off notifications on your phone or for the EMA app
14. You turned off Bluetooth detection
15. You turned off location detection (for the phone or the EMA app)
16. None of the above

[Display list from #2]

1. Yesterday, were you within 15 feet of any of these people (for at least 15 minutes) when you did not have your phone on you?
   - - [Person 1]
     - [Person 2]
     - [Person 3]
2. Do you think you might have missed a survey yesterday?

0) No

1) Yes

2) I don’t know

[Display IF #6 >0]

1. Did you not complete a survey yesterday because of your drinking? (check all that apply)

0) No

1) Yes, because I missed a notification

2) Yes, because I chose not to respond to a notification

1. Were there times yesterday when you think our survey system was not working as you expected?

0) No

1) Yes

2) I don’t know

[Display if #39 = 1 or 2]

1. Tell us how the system or surveys might not have been working as you expected.

[Open response]

[Display to everyone]

1. Tell us anything else we should know about your day yesterday. [open response]

Instruction

If you need to reach us or need to tell us something important about your responses to this specific survey, please TEXT us at this NUMBER.

PLEASE BE SURE TO CLICK SUBMIT.

**Random Reports and Beacon-Triggered Reports (identical)**

INSTRUCTION: Please answer the following questions to the best of your ability.

1. In the past hour, who have you been around for any length of time?
   - - [Person 1]
     - [Person 2]
     - [Person 3]

[Display IF #1 has Persons endorsed. Present only persons endorsed in #1]

1. In the past hour, who have you been within 15 feet of for at least 15 minutes?
   - - [Person 1]
     - [Person 2]
     - [Person 3]

[Display IF #1 has Persons endorsed. Present only persons endorsed in #1]

1. In the past hour, who did you interact with in person for at least 15 minutes?
   - - [Person 1]
     - [Person 2]
     - [Person 3]

[Display IF #1 has Persons endorsed. Present only persons endorsed in #1]

1. Of the people you were with in the past hour, who was drinking?
   - - [Person 1]
     - [Person 2]
     - [Person 3]

[Display IF #1 has Persons endorsed. Present only persons endorsed in #1]

1. Of the people you were with in the past hour, who was drinking heavily or was drunk?
   - - [Person 1]
     - [Person 2]
     - [Person 3]


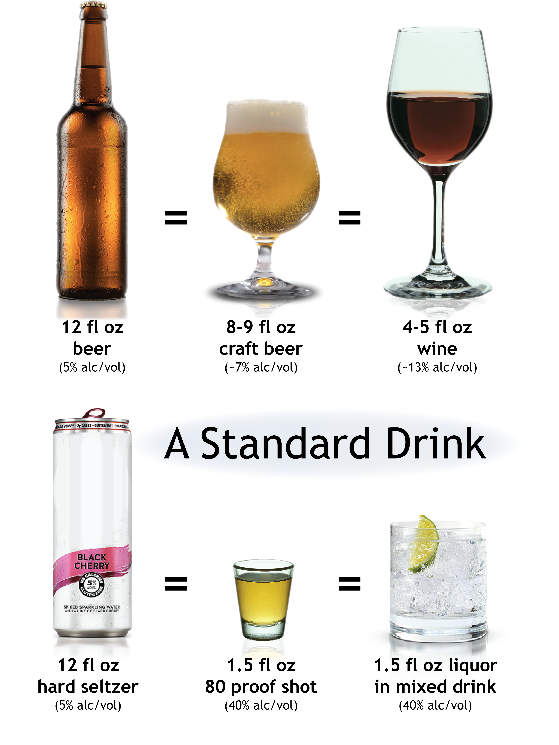


1. How many total standard drinks have you FINISHED so far today? (Don’t count a drink you are drinking now)

(forced choice 0-30)

[Display IF #6 >0]

1. In the past hour, have you been drinking?
   - - 1. No
       2. Yes

[Display IF #7=1 AND IF #1 has Persons endorsed]

1. Who were you with WHILE YOU WERE DRINKING in the past hour?
   - - [Person 1]
     - [Person 2]
     - [Person 3]

[Display IF #7=1 AND IF #8 has Persons endorsed]

1. Of the people you were with in the past hour WHILE YOU WERE DRINKING, who was also drinking?
   - - [Person 1]
     - [Person 2]
     - [Person 3]

[Display IF #7=1 AND IF #8 has Persons endorsed]

1. Of the people you were with in the past hour WHILE YOU WERE DRINKING, who would you say was drunk?
   - - [Person 1]
     - [Person 2]
     - [Person 3]
2. How many people NOT ON YOUR LIST are you around right now?
3. No other people
4. 1
5. 2-4
6. 5-10
7. 11-20
8. 21-50
9. 51-100
10. 101-300
11. 300+

[Display IF #11 =1]

1. Is this person familiar to you?
2. No
3. Yes

[Display IF #11 >1]

1. How many of these people are familiar to you?
2. None
3. Some
4. Half
5. Most
6. All

[Display IF #7=1]

1. In the past hour, where did you consume alcohol? (check all that apply)
2. Your home
3. Friend's place
4. Party
5. Bar/Restaurant
6. Outside
7. Sporting/entertainment event
8. Elsewhere

[Display to everyone]

1. In the past hour, has anyone offered you a drink?

0) No

1) Yes

[Display IF **#15**=1**]**

1. Who offered you a drink? (you can check both answers)

1) Someone on my list

2) Someone NOT on my list

[Display IF #16 =1]

1. Who on your list offered you a drink?

- [Person 1]
- [Person 2]
- [Person 3]

[Display to everyone]

1. In the past hour, has anyone suggested that you should drink? (This may have been someone you weren’t physically around)

0) No

1) Yes

[Display IF #18 = 1]

1. Who suggested you should drink? (you can check both answers)

1) Someone on my list

2) Someone NOT on my list

[Display IF #19 =1**]**

1. Who on your list suggested you should drink?

- [Person 1]
- [Person 2]
- [Person 3]

[Display IF **#7** =1]

1. In the past hour, did anyone refill your drink or get you a new drink?

0) No

1) Yes

[Display IF **#21**=1]

1. Who refilled your drink? (you can check both answers)

1) Someone on my list

2) Someone NOT on my list

[Display IF #22 = 1]

1. Who on your list refilled your drink?

- [Person 1]
- [Person 2]
- [Person 3]

[Display IF #6=0 and #7=0]

1. Is alcohol available to you if you wanted to drink it?
2. No
3. Maybe
4. Yes

[Display IF #6=0 and #7=0]

1. Do you think you will drink today?
2. No
3. Maybe
4. Yes

[Display IF #6 >0 OR #7 = 1)

1. Are you finished drinking today?
2. No
3. Maybe
4. Yes

[Display to everyone]

1. If somebody offered you an alcoholic beverage later today, would you drink it?
2. Definitely not
3. Probably not
4. Probably yes
5. Definitely yes

[Display to everyone]

1. Have you used marijuana today?

0) No

1) Yes

[Display IF #6 >0 OR #28 =1 or 2]

1. How intoxicated do you feel?

0) Not at all intoxicated

1) A little bit intoxicated

2) Moderately intoxicated

3) Very intoxicated

[Display IF #6 >0 and #28 = 1 or 2 and #29 =1, 2, or 3]

1. How much of your intoxication is due to...

1) Only alcohol

2) Mostly alcohol, some marijuana

3) Equally alcohol and marijuana

4) Mostly marijuana, some alcohol

5) Only marijuana

[Display to everyone]

1. Have you used any of these in the past hour? (check all that apply)
2. E-cigarettes/vaping
3. Cigarettes/cigars/cigarillo
4. Nicotine pouch/snus/chew
5. Other drugs
6. None of the above

Instruction

If you need to reach us or need to tell us something important about your responses to this specific survey, please TEXT us at this number NUMBER.

PLEASE BE SURE TO CLICK SUBMIT.

**First Drink Report**

1. Is this your first drink today?
2. No
3. Yes


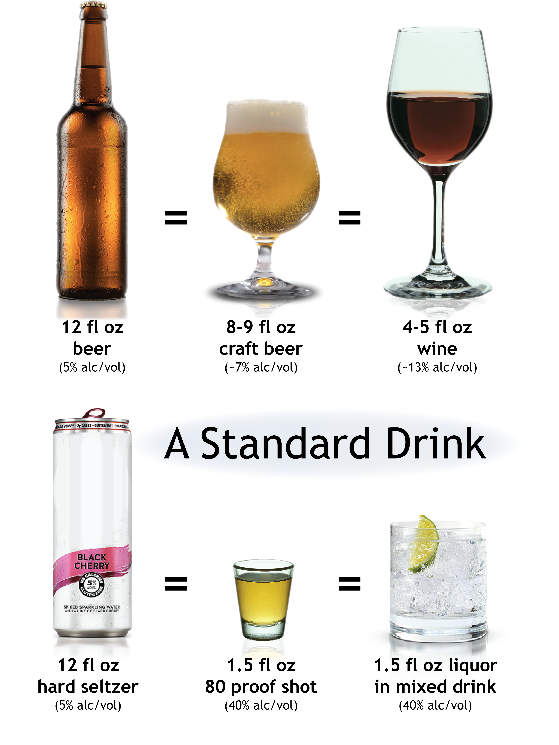


[Display IF #1 = 0]

1. How many total standard drinks have you finished so far today (NOT counting your current drink)?

(Numeric entry)

1. What time did you take the FIRST sip of your FIRST drink?

(Time picker)

1. What was in your FIRST drink? (check all that apply)
2. Beer/Cider/Hard Seltzer
3. Wine
4. Mixed Drink
5. Straight liquor/shot
6. Other
7. How many standard drinks are/were in your FIRST drink?

(Numeric entry)

1. Did anyone give you this FIRST drink without you asking for it?
2. No
3. Yes, someone on my list
4. Yes, someone not on my list

[Display IF #6 = 1]

1. Who gave this drink to you?

- [Person 1]
- [Person 2]
- [Person 3]

1. Who were you with when you started this drink?

- [Person 1]
- [Person 2]
- [Person 3]

[Display IF #8 has Persons endorsed]

1. Of the people you were with when you started this drink, who was drinking?

- [Person 1]
- [Person 2]
- [Person 3]

[Display IF #9 has Persons endorsed]

1. Of the people you were with when you started this drink, who was drinking a lot or was drunk?

- [Person 1]
- [Person 2]
- [Person 3]

1. How many people NOT ON YOUR LIST were you around when you FIRST started drinking?
2. No other people
3. 1
4. 2-4
5. 5-10
6. 11-20
7. 21-50
8. 51-100
9. 101-300
10. 300+
11. Where were you when you started this drink? (select the best option)
12. Your home
13. Friend's place
14. Party
15. Bar/Restaurant
16. Outside
17. Sporting/entertainment event
18. Elsewhere
19. Did you use marijuana WITHIN THE THREE HOURS before you started this FIRST drink?
20. No
21. Yes

[Display IF #13 = 1]

1. Were you under the effects of marijuana when you started your FIRST drink?
2. No
3. Yes
4. Did you use any of these WITHIN THE THREE HOURS before you started this FIRST drink? (check all that apply)
5. E-cigarettes/vaping
6. Cigarettes/cigars/cigarillo
7. Nicotine pouch/snus/chew
8. Other drugs
9. None of the above

**FOLLOW-UP SESSION (INDEX PARTICIPANTS)**

**MEASURES**

1. Protocol debrief items (Self-Administered)
2. Modified System Usability Scale (Self-Administered)
3. Semi-structured Interview (Interviewer Administered)

We would like to know more about your experiences completing surveys on your phone. Please answer the following questions.

1. How easy was it to access and complete the surveys using the Smartphone app?  [0=not at all easy, 3=moderately, 6=very easy]
2. Did you think the length of the morning survey was:  [0=too short, 3=the right length, 6=too long]
3. Did you think the length of the prompted surveys was:  [0=too short, 3=the right length, 6=too long]
4. Did you think the length of the first drink survey was:  [0=too short, 3=the right length, 6=too long]
5. How difficult was it to determine whether friends were closeby? [0=not at all difficult, 3=moderately, 6=very difficult]

Tell us anything more about what it was like reporting about people around you. _________

**Modified System Usability Scale**

The following items are scored on a five-point scale from Strongly Agree to Strongly disagree:

1. I found the app unnecessarily complex.

2. I thought the app was easy to use.

3. I imagine that most people would learn to use this app very quickly.

4. I found the app very awkward to use.

5. I felt very confident using the app.

6. I needed to learn a lot of things before I could get going with this app.

7. The app worked as expected.

8. The app drained my battery.

9. I encountered problems using the app.

10. The app affected other apps on my phone.

11. The app crashed a lot.

12. There were too many survey notifications.

13. I had to re-install the app.

14. I didn't like that I had to change settings on my phone to use the app.

15. I would use this app again in a different research study.

**Semi-Structured Interview**

*This agenda is intended to guide facilitators through the key areas, ensuring that the same content is discussed for each participant. While the list below is used to guide the discussion, it is not a rigid script. This ensures that the facilitators gather data on the same topics for each participant, while also allowing for the flexibility to adapt and clarify questions as needed. Questions may not be asked in this particular order. Facilitators will adapt the questions as needed to clarify issues that arise during the interview.*

“What I would like to do now is to ask you some more questions about your experience in the study, including general questions and impressions you had and also specific questions about our procedures and technology. Is it ok if I get started with that?”

1. Theme: Peer involvement

- In general, how did you feel about your friends being in the study?
- Did you have concerns regarding the privacy of your friends’ participation? What about their responses?
- Did you have concerns that your friends might find out about your responses?
- Did you feel like your friends were monitoring your drinking? How did this affect your behavior? How did this affect your relationship with your friends?
- Did your friends tell you about their study experiences? Were they overall positive or negative about their participation? If you are comfortable telling us, what sorts of concerns did they raise? What did they like?
- There were some times when you interacted with friends during the study that were not listed on the survey when we met at the beginning of the study (i.e., at baseline session on the social network inventory) (*probe specific drinking events/peers*)
- How well do you think the study did at having you identify friends, especially ones you drink with?
- There were some people you named in the survey at the beginning of the study that you never interacted with. Why do you think this was?
- There were some people you named as being friends you often drink with, but you never drank with them during the study. Why do you think this was?
- What could we do to improve the process where we have you identify friends you drink with?

1. Theme: User experience, feasibility, and acceptability

1a. General project experience

- Generally, what are your impressions of the EMA protocol? How challenging? Boring? Stressful?
- What sort of barriers or difficulties did you experience?
- In general, how easy or difficult was it to use the app? What made it easy? What made it difficult?
- Did you have any problems with the app?

1b. Survey content, length, frequency

- In general, did you have any difficulties understanding the questions in the survey?
- Did you have any problems filling in the EMA questions on the phone?
- Were you able to complete surveys in a private way? Were you concerned that others would see your responses?
- Did you feel confident the information collected by the phone would be seen only by the researchers?
- Over the course of a day, you completed a few different types of surveys. What did you think about this – did you find it confusing? What did you like about it? What did you not like about it?
- What did you think about the morning survey– was it too long, too short, just right (length)?
- What about the other kinds of surveys?
- What did you think about the number of times that you were prompted to do a survey? Just right, too much?
- How about the frequency of receiving prompts – were they scheduled too frequently or just right?
- Did the prompts interfere with your activities? Were they disruptive?
- Did you have problems remembering to carry your phone with you every day? Keeping it charged? Why do you think this was? Can you think of changes that could be made to the study that would help with this?
- Did carrying the phone make you behave differently than if you didn’t have it?

1c. Study duration

- What did you think about how long the study lasted -- was 21 days to long? Would 28 days be doable? What is the longest time you think a study like this should last?

1. Themes: Reliability of the app

- Thinking back on the times you didn’t complete a survey, why do you think that was? (*probe: too busy, too much burden, bad timing, didn’t have the phone on them, app wasn’t working properly, didn’t see the notification, forgot to turn it off airplane mode, etc.*)

*If there are particular concerns about the app not catching the beacon Bluetooth signal we will explore with the participant the circumstances of specific events, including where they were (e.g., inside vs. outside)*

1. Overall experience

- Would you be interested in participating in similar studies in the future?
- Would you recommend to others that they participate in a similar study?
- Do you have any suggestions for us about the study (*probe: the protocol, the app, survey items, the Bluetooth beacon, having friends be part of the study, the compensation*)
- Do you think peer proximity technology such as this could help people who are trying to change their drinking? What would be useful?
- Do you think peer proximity technology such as this could help people who are trying to drink in a more safe manner or to minimize alcohol-related consequences (e.g., injury, risky sex)? What would be useful?
- Any other comments or suggestions?
- What other feedback would you have for us about the technology we used?
- What other feedback would you have for us about the participation part of the study?

PEER PARTICIPANT MEASURES

**Follow up peer survey (end of weeks 1,2, 3)**

1. Which kind of beacon do you have?

*1=tag (show image)*

*2=card (show image)*

1. How easy/hard was it to find a place to keep the beacon?

1- Extremely Hard

2- Moderately Hard

3- Somewhat Hard

4- Neither Hard nor Easy

5- Somewhat Easy

6- Moderately Easy

7- Extremely Easy

1. Was it easy/hard to carry the beacon every day?

1- Extremely Hard

2- Moderately Hard

3- Somewhat Hard

4- Neither Hard nor Easy

5- Somewhat Easy

6- Moderately Easy

7- Extremely Easy

Were there any days this past week when you did not have your Bluetooth beacon with you but you were out of your house/residence?

*No/Yes*

*If yes*: please indicate what day(s) and time of day this past week you did not have your Bluetooth beacon with you.

|  | Day (5am-5pm) | Evening (5pm-10pm) | Late night (10pm-5am) |
| --- | --- | --- | --- |
| Day 1 |  |  |  |
| Day 2 |  |  |  |
| Day 3 |  |  |  |
| Day 4 |  |  |  |
| Day 5 |  |  |  |
| Day 6 |  |  |  |
| Day 7 |  |  |  |

What times this past week did you spend time with (*friend name*)?

|  | Day (5am-5pm) | Evening (5pm-10pm) | Late night (10pm-5am) |
| --- | --- | --- | --- |
| Day 1 |  |  |  |
| Day 2 |  |  |  |
| Day 3 |  |  |  |
| Day 4 |  |  |  |
| Day 5 |  |  |  |
| Day 6 |  |  |  |
| Day 7 |  |  |  |

What times this past week were you drinking with (*friend name*)?

|  | Day (5am-5pm) | Evening (5pm-10pm) | Late night (10pm-5am) |
| --- | --- | --- | --- |
| Day 1 |  |  |  |
| Day 2 |  |  |  |
| Day 3 |  |  |  |
| Day 4 |  |  |  |
| Day 5 |  |  |  |
| Day 6 |  |  |  |
| Day 7 |  |  |  |

1. What other feedback do you have for us? _____________

**Peer Interview (end of Week 3)**

“Hello __________, thank you for agreeing to talk about your experience participating in our research. Thank you for participating. Is it ok if I just get started and ask you some questions?”

1.What was your initial reaction when your friend (insert name) asked you to participate in this study? What increased your interest–why did you agree to participate? What sorts of hesitations or concerns did you have?

2.Did you have trouble finding a place to keep the beacon? Did you have problems carrying the beacon every day? What did you think of the beacon form and size? Can you think of changes that could be made to the study that would help with this?

3.Were there things that you didn’t happen like you expected? Ways we could prepare participants like you better? Things that you know now that are important for us to tell people up front?

4. Did anybody else know you were part of the study? What sorts of things did they know? How did they find out? Were you uncomfortable with that?

5. Did being part of the study change anything about your behavior? Why was that? (probe: carrying the beacon, feeling like being monitored)

6. Did being part of the study change anything about your interactions with your friend? What sorts of things were different?

7. If you had been asked to download an app on your phone that would have the same function as the beacon would you have been more or less likely to participate (or no different)?

8. Do you think the monetary compensation for participating was adequate?

9. What other feedback would you have for us about the study?

(When done) “Thank you that is all the questions I have. Do you have any questions for me?”

Index Participant Reasons for Decline Measure

If you don’t mind telling us, why are you not interested in participating in the study? Please check all that apply.

- I am not interested in participating in a research study
- I don’t have enough time/study is too time consuming
- I am worried about potentially identifying information being gathered
- The compensation (payment) is too low.
- I don’t understand the study
- I am afraid that I might not be able to stop participating
- I don’t trust that the research won’t be tracking me
- I don’t think I have friends who would be willing to participate
- Something else: (text box)

Peer Participant Reasons for Decline Measure

If you don’t mind telling us, why are you not interested in participating in the study? Please check all that apply.

- I am not interested in participating in a research study
- I don’t have enough time/study is too time consuming
- I am worried about potentially identifying information being gathered
- The compensation (payment) is too low.
- I don’t understand the study
- I am afraid that I might not be able to stop participating
- I don’t trust that the research won’t be tracking me
- I don’t feel close enough to the participant who asked
- I am worried that my friend would report on my alcohol use
- Something else: (text box)
